# Palliative Radiation Therapy without Chemotherapy for a Patient with Monomorphic Epitheliotropic Intestinal T Cell Lymphoma: A Case Report

**DOI:** 10.1089/pmr.2022.0011

**Published:** 2022-11-03

**Authors:** Takeaki Kusada, Takuro Ariga, Kaori Kina, Yohei Okubo, Masaya Kiyuna, Tsutomu Kadekaru, Takeshi Tomiyama, Hisashi Kamiya, Masaki Gibo, Akihiro Nishie

**Affiliations:** ^1^ Department of Radiology, Okinawa Red Cross Hospital, Naha, Japan.; ^2^ Department of Radiology, Graduate School of Medicine, University of the Ryukyus, Nishihara, Japan.; ^3^ Department of Hematology, Okinawa Red Cross Hospital, Naha, Japan.; ^4^ Department of Surgery, Yuuai Medical Center, Tomigusuku, Japan.; ^5^ Department of Pathology, Yuuai Medical Center, Tomigusuku, Japan.; ^6^ Department of Neurosurgery, Nanbu Tokushukai Hospital, Yaese, Japan.; ^7^ Department of Surgery, Okinawa Red Cross Hospital, Naha, Japan.

**Keywords:** EATL, MEITL, radiation therapy, radiotherapy, RT

## Abstract

Monomorphic epitheliotropic intestinal T cell lymphoma (MEITL), which used to be known as type 2 enteropathy-associated T cell lymphoma, is a rare lymphoma and is generally treated with chemotherapy. However, the MEITL prognosis is poor, and intestinal lymphoma including MEITL has the risk of bowel perforation not only at presentation but also during chemotherapy. A 67-year-old man was diagnosed with MEITL after presenting in our emergency room with bowel perforation. He and his family did not opt for the administration of anticancer drugs because of the risk of bowel perforation. However, they wanted the patient to receive palliative radiation therapy without chemotherapy. This treatment shrunk the tumor size without causing severe complications or decline in the quality of life, until he accidentally died due to traumatic intracranial hematoma. Considering the potential efficacy and safety of this treatment, it should be studied in more patients with MEITL.

## Introduction

Monomorphic epitheliotropic intestinal T cell lymphoma (MEITL) is a rare lymphoma that used to be known as type 2 enteropathy-associated T cell lymphoma (EATL). Since 2017, the World Health Organization has distinguished MEITL from EATL considering its association with celiac disease.^1^ Therefore, the clinical experience of MEITL has not only been reported as MEITL but also as type 2 EATL, EATL, and peripheral T cell lymphoma.^1–11^ The annual incidence of EATL, including MEITL (EATL/MEITL), was reported as 0.5–1.4 per million individuals, with MEITL constituting 34% of the total cases.^2–4^

EATL/MEITL is generally treated with chemotherapy, such as cyclophosphamide, doxorubicin, vincristine, and prednisone (CHOP). Although chemotherapy with other treatments occasionally achieves long overall survival (OS), several studies have reported that the median OS and failure-free survival (FFS) of EATL/MEITL are not >12 months.^1–11^ Moreover, intestinal lymphoma, including MEITL, has a high risk of bowel perforation not only at presentation but also during chemotherapy.^12^ Considering the poor prognosis, MEITL seems to need a more curative treatment strategy as well as palliative treatment strategy.

Radiation therapy (RT) is a useful treatment in palliative management, and three-dimensional conformal RT (3D-CRT) enables us to quantitatively evaluate the complication risk.^7,13–15^ However, the treatment efficacy and safety for MEITL have been insufficiently studied because RT is rarely administered to patients with EATL/MEITL.

In this study, we reported a patient with MEITL who was treated with RT without chemotherapy, whose history will play an important role in the palliative treatment strategy for MEITL.

## Case Description

**FIG. 1.**
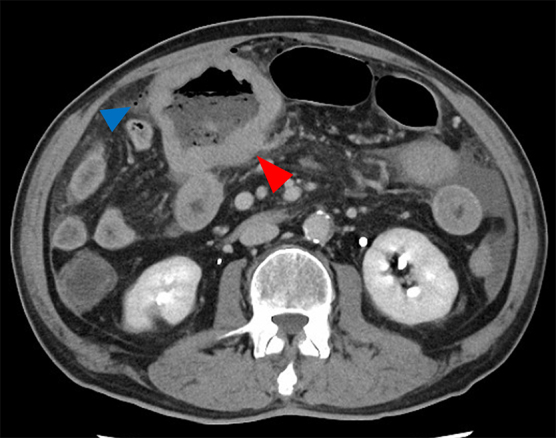
Contrast-enhanced CT at the first medical examination. The red arrow indicates dilatation and thickening of the wall of the intestine. The blue arrow indicates free air and mesenteric fat stranding in the abdomen. CT, computed tomography.

A 67-year-old man presented to our emergency room with the complaint of abdominal pain. Computed tomography (CT) revealed intestinal dilatation, intestinal wall thickening, free air, and mesenteric fat stranding in the abdomen (Fig. 1). We decided to perform emergency surgery to resect the small bowel around the perforation. During surgery, a perforation was detected in the jejunum, and the thickened wall around the perforation was suspected to be a tumor. A part of the thickening wall attached to the transverse colon and the greater omentum was unresectable; therefore, these were left unperturbed. Under microscopic examination, the tumor tissue around the perforation and a part of the greater omentum transmurally contained atypical round cells of different nuclear sizes and narrow polytopes.

The major parts of the tumor showed CD8 immunoreactive cells, and the minor parts presented CD4 staining. We suspected EATL and consulted with an external institution, where additional immunophenotype was determined. Atypical cells were stained positively for UCHL-1 (focal), CD3, CD5 (partial), CD8, CD4 (focal), CD56, and TIA-1 and negatively for CD20, CD79a, CD10, and κ and λ. A small number of cells were positive for EBER. Lymphoid cells were positive for CD103 in the mucosal layer but negative in the submucosa. Using the results of the examinations, the patient was diagnosed with MEITL.

After surgery, we inquired another external institution to perform 18F-fluorodeoxyglucose positron emission tomography (PET)/CT for staging. PET showed extremely high intensity areas in the tumor around the scar of the small bowel. Moreover, it showed extremely high intensity areas in the gastrointestinal tract and slightly high intensity areas in the tumor of the lower lobe of the right lung. Although the lesions in the sigmoid and right lung were not diagnosed by biopsy, we clinically diagnosed metastases using the CT images. According to the results of the aforementioned examinations, the patient was diagnosed with stage IV MEITL based on the Ann Arbor classification. We explained the treatment methods and prognosis, and the patient refused to administer anticancer drugs due to the risk of bowel perforation. Thus, we proposed palliative RT without chemotherapy.

In RT, a 10 MV X-ray was selected, and 40 Gy in 20 fractions was irradiated at each lesion using 3D-CRT (CLINAC iX^®^; Varian Medical Systems, Palo Alto, CA). We defined the aforementioned tumors and bowel thickening in the small intestine, sigmoid, and the lower lobe of the right lung on CT at expiration as gross tumor volumes (GTVs). Only high intensity areas in PET scans without tumor or bowel thickening were not considered as GTVs. In the abdomen, the total area of GTVs and the areas where tumor invasion and adhesion were suspected on CT were defined as clinical target volumes (CTVs). In the right lung, the area of CTV was believed to be similar to that of GTV.

**FIG. 2.**
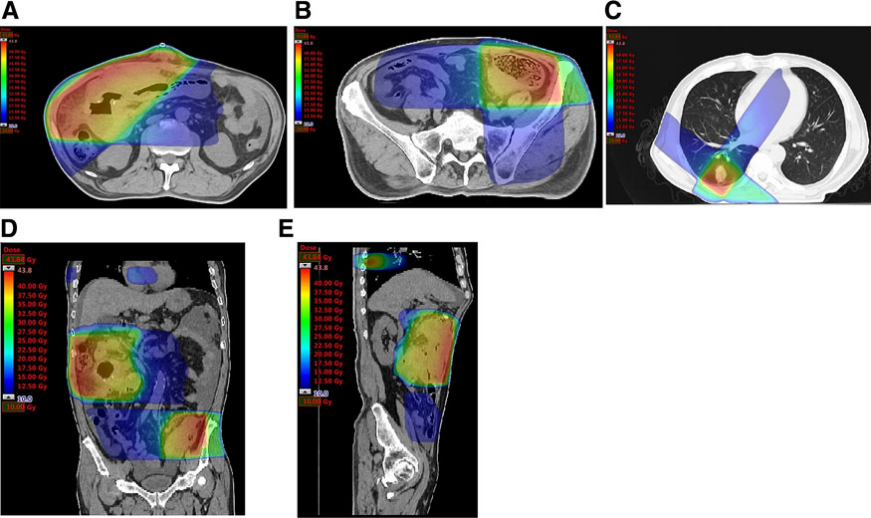
Dose distribution for RT. **(A–C)** Axial image at small bowel, sigmoid, and lower lobe of the right lung lesions, **(D)** coronal image, and **(E)** sagittal image. Color areas denote distribution over 10 Gy. RT, radiation therapy.

The irradiated areas were selected by fitting the planning target volumes, which were obtained by adding each CTV to a 1 cm margin for internal and setup errors. Furthermore, overlap between the areas was avoided, and the areas were irradiated by two non-opposite fields with wedges (Fig. 2). On each radiation day, his position was set up according to the bone image on board, and the sites were irradiated at expiration. Table 1 shows the dose volume histogram parameters. The prescription dose was determined as the minimum dose required for local control. RT started 78 days after surgery for perforation, and he underwent RT by commuting from his home.

**Table 1.** Dose Volume Histogram Parameters of Radiation Therapy

	Dose (Gy)	Volume (cc)
Clinical target volume in small intestine		
Max dose	43.8	
Min dose	19.5	
Mean dose	38.2	
Clinical target volume in sigmoid		
Max dose	43.8	
Min dose	32.5	
Mean dose	40	
Clinical target volume in lungs		
Max dose	42.3	
Min dose	35.1	
Mean dose	39	
Planning target volume in small intestine		
Max dose	43.8	
Min dose	3.6	
Mean dose	36.8	
Planning target volume in sigmoid		
Max dose	43.8	
Min dose	15	
Mean dose	38.6	
Planning target volume in lungs		
Max dose	43.8	
Min dose	23.4	
Mean dose	36.7	
Small intestine^a^		
Max dose	43.8	
V15		608
Bowel bag^b^		
V45		0
Liver		
Mean dose	2.8	
Kidneys		
V12		0.4%
Lungs^c^		
Mean dose	3.5	
V20		4.1%

^a^ Small intestine contained duodenum, jejunum, and ileum.

^b^ Bowel bag contained entire potential space within peritoneal cavity.

^c^ A part of right and left lungs were not contained in computed tomography taken for radiation therapy.

During RT, he did not show severe complications; however, he had grade 1 (anemia, hyponatremia, and anorexia) and grade 2 (alanine and aspartate amino transferase increased, whereas hypoalbuminemia and white blood cell decreased) complications, which were evaluated using common terminology criteria for adverse events.

**FIG. 3.**
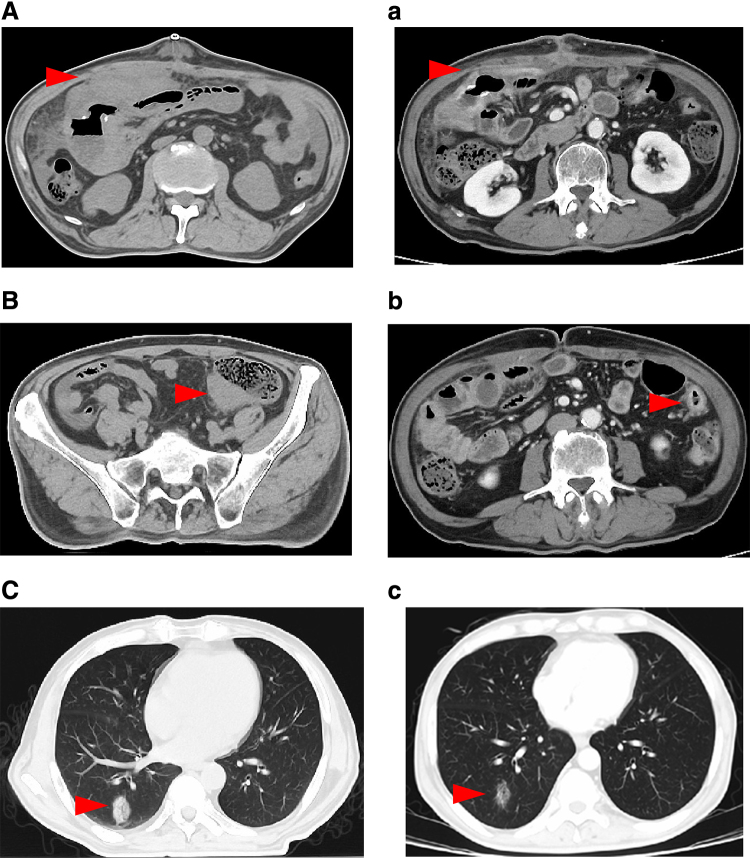
CT before RT **(A–C)** and contrast-enhanced CT just after RT **(a–c)**. The red arrows indicate each individual lesion. A/a, small bowel; B/b, sigmoid; C/c, lower lobe of the right lung.

On the final radiation day, immediately after the radiation, CT showed shrinkage of all tumors (tumor size before and after RT were as follows: around the small bowel, 10.8 × 6.1 × 13.1 cm and 6.8 × 4.2 × 2.8 cm; sigmoid, 2.7 × 2.7 × 10.5 cm and 1.7 × 2.0 × 4.9 cm; and right lung, 3.0 × 3.1 × 2.0 cm and 3.0 × 1.5 × 1.3 cm, respectively) (Fig. 3). The patient's activities of daily living had not decreased; thus, he could walk and consume a normal diet. After RT, he was followed up weekly at our hospital. The serum soluble interleukin-2 receptor level tended to decrease (before RT, 1352 U/mL; a week after RT, 979 U/mL; and five weeks after RT, 797 U/mL). He did not show severe complications but had grade 1 (anemia and hyponatremia) and grade 2 (decrease in white blood cell count and hypoalbuminemia) complications.

At 39 days after RT, he was found lying unconscious and smelled of alcohol. He was taken to the closest emergency hospital, where head CT revealed an acute subdural hematoma and fracture of the left parietal bone. Furthermore, chest CT was conducted, which showed slight shrinkage of the tumor in the right lung (2.2 × 1.3 × 1.0 cm) and no free air in the upper abdomen. Conservative treatment was administered, as his condition implied that surgery for intracranial hematoma would be intolerable. After another traumatic hematoma that occurred in the left frontal brain lobe, he died 44 days after the end of RT.

## Discussion

RT without chemotherapy worked effectively and safely as a palliative treatment in a patient with MEITL.

Although chemotherapy is the most effective treatment for EATL/MEITL, the median OS and FFS of EATL/MEITL are not >12 months.^1–11^ Sieniawski et al also reported that most patients with EATL/MEITL died because of disease progression or treatment complications.^4^ According to the aforementioned poor prognosis, MEITL seemed to need a palliative treatment strategy, including RT.

A limited number of cases have been reported on the treatment outcomes with RT for MEITL. Till June 26, 2022, only 13 articles were found on PubMed using the following keywords: “monomorphic epitheliotropic intestinal T-cell lymphoma” or “MEITL,” “enteropathy-associated T-cell lymphoma” or “EATL,” and “radiation therapy” or “radiotherapy.” To the best of our knowledge, although there are some reports on RT without chemotherapy for gastrointestinal non-Hodgkin's lymphomas, such as the report by Watanabe et al,^16^ no other report details RT without chemotherapy for MEITL.

Nonetheless, some articles have shown an adjuvant or palliative effect of RT with chemotherapy for MEITL. Petrich et al reported a population-based analysis of 8802 patients with peripheral T cell lymphoma, where RT omission caused a risk of inferior OS in univariate analysis.^5^ Novakovic et al reported six patients with EATL and peripheral T cell lymphoma who were treated with CHOP and RT as the first line of treatment^6^; all of them achieved a temporary complete response. Nato et al reported that the activities of daily living improved during the whole brain RT in a patient with MEITL who suffered a central nervous system relapse after chemotherapy and cord blood transplantation.^7^ Delabie et al reported that a large tumor of EATL of >5 cm often results in complications such as perforation^3^; therefore, local control by RT may play an important role not only as a palliative treatment but also as a curative treatment due to the risk of bowel perforation.

MEITL has a high risk of bowel perforation at presentation. Ma et al reported that the incidence of initial bowel perforation was high in patients with T/NK-cell lymphomas, including five patients with EATL with aggressive B cell lymphomas.^8^ Tse et al reported that 13 of 38 patients with type 2 EATL had initial bowel perforation.^9^ Novakovic et al reported that 5 of 10 patients with EATL had intestinal perforation at diagnosis.^6^ Similarly, in our patient, MEITL was discovered after the occurrence of bowel perforation.

Moreover, intestinal T cell lymphoma presents the risk of repeated perforation. Yamamoto et al reported a case of intestinal T cell lymphoma in which small intestine perforation occurred seven days after a surgery for sigmoid perforation.^17^ Sun et al suggested that one of the reasons for repeated perforation is the histopathological feature of intestinal T cell lymphoma and that the lymphomatous infiltrate is diffuse and multifocal in the wall of the intestine.^18^ Considering these reports of repeated perforations, the risk of repeated perforation might also have occurred in this study.

Alternatively, Vaidya et al reported that of 100 perforation events, 49 occurred during treatment with chemotherapy,^12^ and patients with intestinal lymphoma simultaneously had a simultaneous risk of perforation during chemotherapy. In a pathological review of specimens removed at surgery for 94 bowel perforations, although 69 contained tumors, the remaining 25 did not show any evidence of lymphoma. Fourteen of those without tumors had acute inflammation and tumor necrosis due to chemotherapy and two of them presented with radiation enteritis. Although the bowel perforation frequency in each treatment remains unclear, the aforementioned report seemed to show that the bowel perforation risk in RT for MEITL is lower than that in chemotherapy.

Some reports on the dose of organs at risk (OAR) can be referred to predict RT complications. Marks et al reported the probability models of normal tissue complications. As the parameters of bowel dose, they introduced the volume of receiving >15 Gy (V15) in individual small bowel loops and V45 in the entire potential space within the peritoneal cavity were the parameters for acute severe small bowel complications.^14^ Zheng et al reviewed various reports on radiation dose for the small bowel, including the duodenum, and introduced V15, V40, V50, and V55.^15^

Because the treatment goal was local control without severe complication (palliative treatment), paying attention to the aforementioned dose parameter, we determined the prescription dose of 40 Gy as the minimum dose for peripheral T cell lymphoma without chemotherapy as per the National Comprehensive Cancer Network guidelines.^10^ Sieniawski et al reported that 8 of 54 patients with EATL/MEITL had severe gastrointestinal complications, including perforation during chemotherapy.^4^ Accordingly, we believe that the risk of complication using RT in our case was lower than that using chemotherapy because we could retain V40, V45, V50, and V55 and control the bowel lesions.

Finally, we believe that RT for MEITL metastasis should be positively selected. Bhatlapenumarthi et al reported that shortness of breath occurred in a patient who had lung metastasis of MEITL.^11^ Patterson et al reported the potential role of local control by RT for metastatic lesions of gastrointestinal stromal tumors to palliate symptomatic metastases as well as to prolong survival as surgery.^13^ Considering the complication-associated metastasis and the RT efficacy, we believe that all sites of metastasis should be irradiated if RT without chemotherapy can control those in the acceptable dose of OAR.

The limitation in this case is the short observation period after RT due to accidental death. In addition, we could not decide the start date for RT before surgery for perforation.

Thus, in palliative RT without chemotherapy for MEITL, the duration of this treatment effect and the risk of severe chronic complications associated with it have not yet been determined. Considering that adjuvant RT for colon cancer is started approximately one month after surgery, as observed in the report by Martenson et al,^19^ it is necessary to determine whether we can start palliative RT safely one month after surgery. Thus, further case reports and clinical studies are needed.

In conclusion, palliative RT without chemotherapy worked without severe complications, including bowel perforation, in our patient. Considering the potential efficacy and safety of this treatment, it should be studied in more patients with MEITL.

## Abbreviations Used

3D-CRT: three-dimensional conformal RT
CT: computed tomography
CTVs: clinical target volumes
EATL: enteropathy-associated T cell lymphoma
FFS: failure-free survival
GTVs: gross tumor volumes
MEITL: monomorphic epitheliotropic intestinal T cell lymphoma
OAR: organs at risk
OS: overall survival
PTVs: planning target volumes
RT: radiation therapy
